# Aspects descriptifs du VIH/SIDA chez les sujets âgés de 50 ans et plus suivis au Centre de Traitement Agréé de Bafoussam - Cameroun

**Published:** 2012-08-14

**Authors:** François-Xavier Mbopi-Kéou, Lucienne Dempouo Djomassi, Francisca Monebenimp

**Affiliations:** 1 Laboratoire National de Santé Hygiène Mobile, Ministère de la Santé Publique, Yaoundé, Cameroon et Faculté de Médecine et des Sciences Biomédicales, Université de Yaoundé I, Yaoundé, Cameroun; 2 Direction de la Lutte contre la Maladie, Ministère de la Santé Publique, Yaoundé, Cameroun; 3 Centre Hospitalier Universitaire de Yaoundé et Faculté de Médecine et des Sciences Biomédicales, Université de Yaoundé I, Yaoundé, Cameroun

**Keywords:** VIH, sujets âgés, antirétroviraux, survie, Cameroun, HIV, elderly, antiretroviral, survival, Cameroon

## Abstract

**Introduction:**

La littérature scientifique dispose de très peu de données relatives à l’épidémiologie du VIH chez les sujets âgés en Afrique subsaharienne. Au Cameroun, les caractéristiques épidémiologiques de l'infection par le VIH chez les sujets âgés de 50 ans et plus ne sont pas documentées.

**Méthodes:**

Dans une étude de cohorte rétrospective et une enquête transversale, nous avons comparé les caractéristiques clinico-biologiques et la survie post thérapeutique des patients âgés de 50 ans et plus, sous traitement antirétroviral au Centre de Traitement Agrée de Bafoussam - Cameroun, aux adultes plus jeunes.

**Résultats:**

L’âge moyen était de 39 ans, les extrêmes étant 17 et 88 ans. Les sujets âgés de 50 ans et plus représentaient 14,1% des cas. Les plus âgés étaient moins bien informés sur les modes de transmission du virus (p = 0,04). Leur séropositivité au VIH était le plus souvent découverte au décours d'une infection opportuniste (p = 0,02). La fréquence de comorbidité était significativement plus élevée chez les personnes âgées de 50 ans et plus (p < 10-5). Nous n'avons pas retrouvé une association statistiquement significative entre l'observance thérapeutique et l’âge (p = 0,83). La survie post-thérapeutique n’était pas significativement liée à l’âge (p = 0,81).

**Conclusion:**

Les sujets âgés ne sont pas à l'abri du VIH. La promotion du dépistage et les programmes d’éducation sanitaire relatifs au VIH/SIDA devraient être renforcés au sein de cette communauté déjà affaiblie par le poids de l’âge, afin de réduire l'incidence du SIDA et de leur assurer prise en charge précoce.

## Introduction

Avant l'avènement des antirétroviraux, la survie médiane des patients atteints de SIDA était limitée à 11 mois [1]. Le traitement antirétroviral (TAR) hautement actif a amélioré le pronostic [3–5] et la survie [6, 7] des personnes infectées par le VIH. Les malades vivent plus longtemps et dans le temps, une recrudescence de nouvelles infections au VIH dans les tranches d’âge avancé a été observée. Aux Etats-Unis, la proportion de personnes infectées par le VIH/SIDA âgées de 50 ans et plus a plus que doublé entre 1994 et 2005 : 10,4% en 1994 [8], 11% en 1996 [9], 17% en 2001 et 24% en 2005 [10]. En France, la proportion de nouvelles infections chez les plus de 50 ans est passée de 16,8% en 2005 [11] à 18% en 2006 [12].

Les rapports sexuels constituent la principale voie de contamination chez les sujets âgés [13–15] ; pourtant, ils ne sont pas communément considérés comme sexuellement actifs [16]. Les résultats des études portant sur leur réponse à la trithérapie antirétrovirale sont contradictoires. Pour certains auteurs, leur survie post-thérapeutique est plus brève [17, 18], alors que pour d'autres, elle n'est pas influencée par l’âge [19–22].

L'allongement de l'espérance de vie des personnes vivant avec le VIH sous TAR n'explique pas totalement l'augmentation de nouvelles infections au VIH chez les personnes âgées. D'autres facteurs y interviennent de façon concomitante, au nombre desquels: l'allongement de la vie sexuelle par l'utilisation fréquente des médicaments de la dysfonction érectile ; ils ont en effet été associés à des comportements sexuels à risque [23] ; la méconnaissance des risques de transmission du VIH par les personnes âgées [24] ; la faible utilisation des préservatifs par ces individus [25] ; le manque de programme d’éducation et de prévention ciblant les plus âgés [26].

Pourtant, cette population ne fait l'objet d'aucun des 25 indicateurs de surveillance de l’éradication du VIH sur la planète utilisés par l'ONUSIDA [27]. Dans les pays du Sud, les données de l'OMS sur l’épidémiologie du VIH se limitent à 49 ans [28].

Au Cameroun, la séroprévalence à VIH a été estimée à 5,5%. L’épidémie touche de façon disproportionnée les femmes et la tranche d’âge la plus affectée est de 15-24 ans. Les caractéristiques épidémiologiques de l'infection par le VIH chez les sujets âgés de 50 ans et plus ne sont pas documentées [2].

Aussi, nous nous proposons d’étudier les aspects épidémiologiques, cliniques et la survie chez les sujets âgés de 50 et plus lors de la mise sous TAR au Cameroun. Plus spécifiquement, il s'agit pour nous de: décrire les caractéristiques cliniques et biologiques de l'infection par le VIH chez les sujets âgés de 50 ans et plus en début de TAR; déterminer leur niveau d'observance du TAR; décrire leur survie post thérapeutique ; comparer les caractéristiques cliniques de l'infection par le VIH, le délai de prise en charge, l'observance thérapeutique et la survie post thérapeutique entre les sujets âgés de 50 ans et plus et les plus jeunes.

A cet égard, nous faisons les hypothèses que: les sujets âgés séropositifs au VIH présentent des caractéristiques épidémiologiques et cliniques spécifiques ; la réponse à la trithérapie antirétrovirale n'est pas liée à l’âge [21, 22] et l'observance au traitement est meilleure chez les plus âgés [29, 30].

## Méthodes

### Schéma d’étude

Une étude de cohorte rétrospective ainsi qu'une enquête transversale ont été réalisées dans le Centre de Traitement Agrée (CTA) de Bafoussam, structure de prise en charge des personnes vivant avec le VIH (PVVs) abritée par l'hôpital de référence de la région ouest – Cameroun (Hôpital Régional de Bafoussam) et servant de tutelle et de référence aux autres Unités de Prise en Charge de PVVs (UPEC) siégeant au sein des 19 hôpitaux de district de la région. Sa file active en date du 31 Décembre 2009 était de 2049 malades sous TAR.

### Population d’étude

Notre échantillon, constitué à partir des registres comprenait tous les patients séropositifs pour le VIH âgés de 16 ans ou plus, mis sous traitement antirétroviral à partir du 1er Janvier 2007 et suivis depuis au moins 3 mois au CTA de Bafoussam jusqu'au 31 Décembre 2009. L’étude transversale a été réalisée dans un sous-échantillon de patients venus procéder au renouvellement mensuel de leur ordonnance entre le 22 Février et le 24 Mars 2010, et ayant donné leur accord de participation par un consentement éclairé.

### Données recueillies

Un questionnaire a été construit pour recueillir des données sur les caractéristiques socio-démographiques (date de naissance, sexe, statut matrimonial) des patients à l'inclusion, les caractéristiques cliniques et biologiques des patients en début du TAR, le moment de survenue des infections opportunistes (IO), l'existence d'autres affections non liées au déficit immunitaire, le statut vital à partir des registres du comité thérapeutique, de la pharmacie et des dossiers médicauxsur la base de l'identifiant attribué à chaque patient au commencement du TAR.

Un autre questionnaire s'intéressait aux connaissances relatives à la transmission et la prévention du VIH, aux comportements sexuels et pratiques à risque età l'observance déclarée des 24 dernières heures (Boite 1). La mesure de l'observance a été évaluée sur les 24 dernières heures afin de limiter l'effet d'un éventuel biais de mémorisation. Pour ce dernier, des entretiens ont été conduits par un enquêteur préalablement formé en mode face à face dans les locaux de la salle de « counselling » ou le bureau d'aide à l'observance afin de garantir la confidentialité. L'entretien était conduit en Français et/ou en langue maternelle.

### Analyse statistique

Afin d'assurer une bonne démarcation entre les deux groupes et de permettre des comparaisons avec des études antérieures [20, 31–33], les sujets ont été repartis en deux groupes en fonction de l’âge du patient au moment de la mise en route du TARV : les personnes âgées de 16 à 40 ans (G1) et les personnes âgées de 50 ans et plus (G2).

L’échantillon a été décrit dans son ensemble. Les caractéristiques des groupes 1 et 2 ont ensuite été comparées avec les tests de Khi2 ou de Fisher pour les variables qualitatives et l'analyse par ANOVA ou le test non paramétrique de Mann Whytney/Wilcoxon (quand les variances n’étaient pas homogènes) pour les variables quantitatives. Le degré de signification était fixé à 5%.

Le niveau général de connaissance était apprécié par un score, sommant les différents items évoqués dans les rubriques connaissance sur les modes de transmission et connaissance sur la prévention du VIH. Une note de 1/1était attribuée pour chaque réponse juste. Ce score était étalonné sur une échelle à trois grades : faible, moyen et bon (**Annexe (PDF 68Kb)**).

En ce qui concerne l'analyse de survie, la date d′origine était la date de mise en œuvre du TAR. La date de dernières nouvelles était définie par la date du dernier retrait des ARV à la pharmacie pour les sujets perdus de vue à 3 mois ou plus et la date de décès le cas échéant. La date de point était fixée au 31 Décembre 2009. Les cas de transfert ont été censurés à droite à la date de leur transfert. Le test de Log Rank et le modèle des risques proportionnels de Cox ont été utilisés pour comparer la survie entre les tranches d’âge. L'analyse a été réalisée pour les facteurs de risques recueillis dans les dossiers médicaux : sexe, début du traitement au moment de la gratuité des ARV (en vigueur depuis le 05/05/2007), le stade immunologique [34] et le stade clinique [35] en début de TAR. La saisie des données et l'analyse statistique ont été réalisées avec les logiciels EpiInfoTM version 3.5.1 et SAS 9.1 version française.

## Résultats

### Caractéristiques générales des sujets de la cohorte (Figure 1)

**Figure 1:**
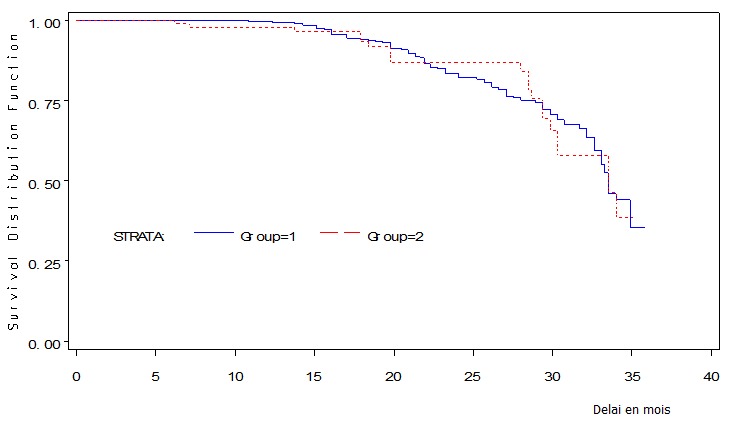
Diagramme de flux de la file active des patients mis sous TAR de Janvier 2007 à Décembre 2009 et suivis au Centre de Traitement Agrée de Bafoussam, Cameroun

L’âge moyen était de 39 ans, les extrêmes étant 17 et 88 ans. Les sujets âgés de 50 ans et plus représentaient 14,1% des cas. Le sex-ratio était de 2,7 femmes pour 1 homme. Les femmes infectées étaient significativement plus jeunes que les hommes (en moyenne 38 ans vs 43 ans, p < 10^-5^). Le traitement avait été initié avant l'avènement de la gratuité des ARV pour 13,40% sujets à l’étude.

### Analyse comparative stratifiée sur l’âge en début de traitement

Les résultats de cette analyse sont rassemblés dans le Tableau 1.

**Tableau 1:** Caractéristiques selon l’âge des patients infectés par le VIH suivis au Centre de Traitement Agrée de Bafoussam (Cameroun) de 2007 à 2009 – Données des dossiers médicaux

Variables	G1 16 - 40 ans N = 688		40-50 ans N = 384		G2 =50 ans N = 202		p-value ‡
	n	%	n	%	n	%	
**Sexe**							<10-5
Masculin	81	18,1	67	40,1	42	41,2	
Féminin	366	81,9	100	59,9	60	58,8	
Manquant	241		217		100		
**Statut matrimonial**							<10-5
Célibataire	64	18,0	12	07,2	02	02,4	
Divorcé(e) ou en séparation	44	12,3	21	12,6	03	03,7	
En union (mariage, cohabitation)	169	47,3	78	46,7	40	49,4	
Veuf (ve)	80	22,4	56	33,5	36	44,5	
Manquant	331		217		121		
**Stade clinique à l'initiation – CDC** **							<10-3
A	203	47,1	65	32,2	31	31,0	
B	183	42,5	112	55,4	61	61,0	
C	45	10,4	25	12,4	08	08,0	
Manquant	257		182		102		
**Stade immunologique à l'initiation – CDC**							0,41
1 (>500 cellules/mm3)	00	00,0	2	01,0	00	00,0	
2 (200-499 cellules/mm3)	47	10,7	12	05,9	08	07,9	
3 (<200 cellules/mm3)	394	89,3	189	93,1	93	92,1	
Manquant	247		181		101		
**Type de protocole thérapeutique**							0,70
Première ligne	436	97,4	162		101	98,8	
Deuxième ligne	11	02,6	5	03,0	01	01,2	
Manquant	241		217	97,0	100		
**Moment de survenue des IO**							0,02
Avant la découverte du statut sérologique	38	43,2	23	11,1	08	33,4	
Au moment du diagnostic sérologique	16	18,2	11	05,3	11	45,8	
Après la mise sous TARV	34	38,6	174	83,6	05	20,8	
Manquant	600		176		189		
**Affection associée**							10-5
Présence	22	04,9	33	15,9	33	32,3	
Absence	425	95,1	175	84,1	69	67,7	
Manquant	241		176		100		
**Statut au moment de l’étude**							0,24
Vivant	522	75,9	281	73,2	141	69,8	
Décédé	38	5,5	24	6,3	18	8,9	
Transféré	45	6,5	32	8,3	15	7,4	
Perdu de vue	83	12,1	47	12,2	28	13,9	
**Taux de CD4 à l'initiation en cellules/mm3**							0,74
Moyenne (n)	123 (452)	105 (266)	126 (171)				
Médiane	125	104	137				
Intervalle inter- quartile	(68 ; 174)	(39 ; 158)	(50 ; 175)				
Manquant	236	118	31				
**Durée de suivi**							0,33
Moyenne (n)	18,84 (495)	19,87 (285)	19,78 (153)				
Médiane	19,28	20,43	19,74				
Intervalle inter- quartile	(8,95 ; 28,92)	(11,90 ; 28,79)	(12,95 ; 28,66)				
Manquant	193	99	49				

* n= effectif

** CDC: Centers for Disease Control and Prevention; IO: Infection Opportuniste

† VIH: Virus de l'immunodéficience humaine

‡ P-value associée aux tests de comparaison entre G1 G2.

* Données manquantes: en rapport avec la variable et/ou une absence totale d'informations sur le taux des CD4 et le stade clinique simultanément

**Caractéristiques socio-démographiques de l'ensemble des patients:** L’âge médian était de 34 ans dans le groupe G1 (Intervalle interquartile: 30; 37).et de 54 ans dans le groupe G2 (Intervalle interquartile: 51 ; 57). On observait en moyenne 4,5 fois plus de femmes que d'hommes dans le groupe G1, alors que dans le groupe G2, la répartition par sexe approchait l’équilibre avec 41,2% d'hommes et 58,8% de femmes (p < 10^-5^). La fréquence des veufs étaient en moyenne 2 fois plus importante chez les plus âgés et celle des célibataires, 7 fois plus élevéechez les 40 ans et moins (p < 10^-5^).

**Caractéristiques clinico-biologiques de l'ensemble des patients:** Les patients du groupe G1 étaient plus souvent mis sous traitement au stade clinique B, CDC-1993 et ceux du groupe G2 au stade A (p = 0,00).On n'observait cependant pas de différence entre les deux groupes pour ce qui est du stade immunologique à l′initiation (p = 0,41). De façon similaire, les moyennes des lymphocytes CD4 à l'inclusion dans les deux groupes n′étaient pas significativement différentes (p = 0,74). La sérologie HIV était le plus souvent découverte lors de la survenue d′une infection opportuniste chez les plus âgés (p = 0,02).Nous n'avons pas mis en évidence une association statistiquement significative entre l′observance au TAR et l′âge (p = 0,83).Un tiers des 50 ans et plus (32,3 %) avaient au moins une affection associée. La fréquence de co-affections était significativement plus élevée chez les plus âgés (p < 10^-5^). L'hypertension artérielle constituait le premier facteur de comorbidité (54,5%) chez les 50 ans et plus, la dépression chez les moins de 40 ans (22,7%) ; les profils de comorbidité étaient significativement différents entre les deux groupes (p = 0,01). La fréquence d′hospitalisations après la découverte de la séropositivité au VIH n’était pas liée à l′âge (0,7 pour G1, 1,3 pour G2, p = 0,19).

### Connaissances attitudes et pratiques des patients uniquement interrogés (Tableau 2)

Les plus jeunes semblaient mieux informés quant aux modes de transmission du VIH (p = 0,04). On observait par contre pas de différence du niveau de connaissance sur la prévention du virus entre les deux groupes (p = 0,16). Les patients des deux groupes présentaient avant la découverte de leur séropositivité au VIH des comportements sexuels similaires (p = 0,45). Les plus jeunes présentaient plus souvent des antécédents descarifications corporelles (50,1% vs 37,3%, p = 0,03).

**Tableau 2:** Caractéristiques selon l’âge des patients infectés par le VIH suivis au Centre de Traitement Agrée de Bafoussam (Cameroun) de 2007 à 2009 – Données des entretiens

Variables	G1 16 - 40 ans N = 397		40 - 50 ans N = 212		G2 =50 ans N = 117		p-value‡
	n	%	n	%	n	%	
**Niveau de connaissance sur la transmission du VIH†**							0,04
faible	93	24,0	63	30,3	35	34,3	
moyen	55	14,2	36	17,3	22	21,6	
bon	239	61,8	109	52,4	45	44,1	
Non réponse	10		4		15		<10^-4^
**Niveau de connaissance sur la prévention du VIH**							0,16
faible	03	00,8	43	20,7	01	01,2	
moyen	35	09,9	13	06,2	13	16,1	
bon	316	89,3	152	73,1	67	82,7	
Non réponse	43		4		36		<10^-4^
**Observance des 24 dernières heures**							0,83
Observants	336	87,0	176	84,2	90	85,7	
Non observants	50	13,0	33	15,8	15	14,3	
Non réponse	11		5		12		<10^-3^
**Scolarisation ?**							<10^-3^
Aucun ou primaire	151	38,0	99	46,7	83	70,7	
Secondaire	233	58,6	96	45,5	30	25,6	
Supérieur	13	3,4	17	7,8	4	3,7	
Non réponse	0		0		0		

* n= effectif

† VIH : Virus de l'immunodéficience humaine

‡ P-value associée aux tests de comparaison entre G1 G2

### Survie des patients sous traitement (Figure 2)

L'analyse de la survie (présence dans la file active) stratifiée sur l’âge a porté sur 447 sujets appartenant au groupe G1 et 105 au groupe G2. La moyenne des temps de suivi chez les patients du groupe 1 était de 36,24 mois ; celle des sujets du groupe 2 était de 34,76 mois. La moyenne de suivi chez les patients sortis d’étude était de 24,81 mois pour les 16-40 ans et de 23,06 mois pour les = 50 ans (p = 0,44). La survie dans les deux groupes n’était pas significativement différente (p= 0,81).

**Figure 2:**
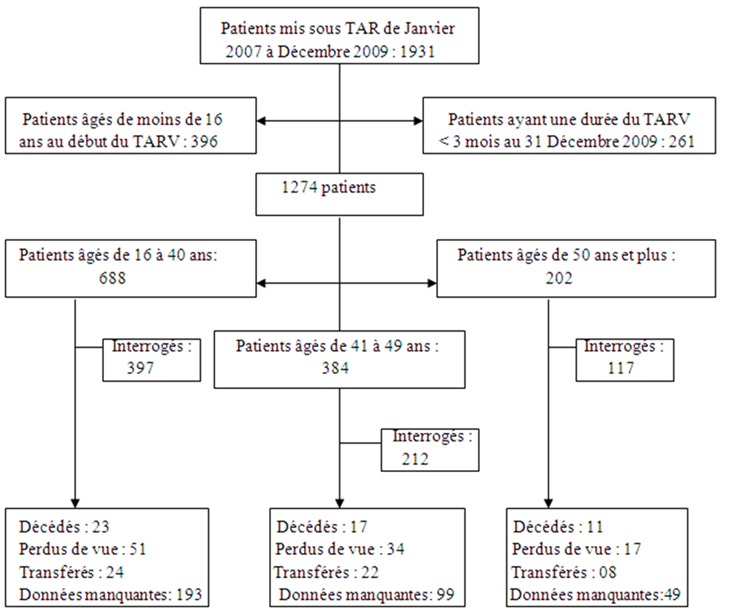
Survie post-thérapeutique selon l’âge : Cohorte des patients infectés par le VIH suivis au Centre de Traitement Agrée de Bafoussam (Cameroun) de 2007 à 2009 (Group-1: 16 - 40 ans; Group-2: 50 ans et plus)

## Discussion

La proportion des sujets âgés de 50 ans et plus était de 14,10%. Cette fréquence serait sous-estimée, la découverte de la séropositivité se faisant en général tardivement dans cette tranche d’âge [36, 37], un bon nombre décèderait précocement [38]. On observait en moyenne deux fois plus de veufs chez les plus âgés et 7 fois plus de célibataires chez les plus jeunes. Les individus qui n'ont jamais été mariés sont plus à risque d'adopter des comportements sexuels à haut risque [39, 40]. La plus grande fréquence de décès du conjoint chez les 50 ans et plus trouverait explication dans le fait que le risque de décès toute cause confondue augmente nettement avec l’âge en raison de la baisse de l'immunité humorale [41] et de la coexistence de plusieurs affections. Dans notre cohorte, les malades des deux groupes présentaient avant la découverte de leur séropositivité des comportements sexuels similaires. Des résultats d’études antérieures ont toutefois suggéré que les sujets âgés sont plus enclin à pratiquer des rapports sexuels non protégés du fait de leur faible niveau de connaissance sur les facteurs de risque et les méthodes de prévention du VIH [25] et de l'utilisation des médicaments de la dysfonction érectile [23]. La moitié de nos patients, plus souvent les jeunes que les plus âgés, avaient subi des scarifications corporelles chez des tradipraticiens, dans des conditions de travail a priori incertaines. L'exercice de cette pratique n'a pas été formellement identifié comme moyen de transmission du VIH au Brésil [42]. En Afrique du Sud, Peltzer notait que bien que la majorité des tradipraticiens fussent bien informés quant aux principales voies de contamination et moyens de prévention du VIH, 4% d'entre eux utilisaient des lames de rasoir sur plus d'un client pour des scarifications [43]. Les plus jeunes apparaissaient mieux informés quant aux risques de transmission du virus par rapport aux plus âgés ; le niveau de connaissances sur la prévention du VIH ne semblait pas lié à l’âge. Maes et Louis ont mis en évidence une corrélation entre la baisse du niveau de connaissance sur le VIH/SIDA et l'augmentation de l’âge [44]. La médiane des lymphocytes CD4 à l'inclusion était de 124 cellules/ mm^3^. Le taux de lymphocytes CD4 constitue un facteur pronostic de l'infection par le VIH. Des faibles taux ont été associés à une progression plus rapide de la maladie aussi bien dans les pays développés [45] que dans les pays à ressource limitée [46].

Les patients du groupe G2 étaient plus souvent mis sous traitement à un stade symptomatique non SIDA par rapport à ceux du groupe 1 qui l’étaient à un stade asymptomatique. Ces résultats suggèrent que le dépistage et donc la prise en charge se font encore tardivement chez les 50 ans et plus par rapport aux plus jeunes, comme l'ont souligné des études antérieures [47, 48]. Un tiers des sujets âgés présentaient au moins une affection associée. L'hypertension artérielle constituait la principale co-affection dans ce groupe (54,5%), la dépression chez les sujets jeunes (22,7%). Magalhães MG et coll dans une étude rétrospective sur la co-morbidité des patients âgés infectés par le VIH observaient que 88,8% d'entre eux avaient au moins une affection associée et 41,4% avaient des antécédents d'hypertension artérielle [49]. L’âge avancé ne semblait pas être associé à une meilleure observance. Ces résultats s'opposent à ceux rapportés par plusieurs auteurs montrant que la meilleure observance était observée chez les plus âgés [50, 51]. Cette situation témoignerait de l'efficacité des programmes d'aide à l'observance et du suivi de proximité mis en œuvre au niveau du CTA. La médiane de survie s’élevait à 33,51 mois, bien supérieure à celle rapportée dans la littérature dans les pays en voie de développement, o[ugrave] elle fluctue autour de 12 mois chez les patients au stade SIDA ou ayant un taux de CD452].La survie dans les deux groupes n’était pas significativement différente (p= 0,89). Certains auteurs ont montré que la survie chez les plus âgés était significativement plus brève et l'ont expliqué par le déficit immunitaire lié à l’âge [17, 18] et par le diagnostic tardif de l'infection chez les sujets âgés [14]. D'autres en revanche, ont retrouvé une similitude dans les réponses immunologique et virologique entre sujets âgés et jeunes [19, 53, 54]. Plus récemment, Tumbarello et al. [20] ont démontré que les plus âgés, bien qu'ayant une infection plus souvent sévère vis-à-vis des plus jeunes sont en mesure de réaliser une réponse clinique identique à ces derniers.

### Limites du travail

La principale limite de ce travail tient lieu du fait que l'analyse n'a porté que sur des données disponibles et est donc sujette à un biais de sélection dont l'effet sur les estimations est difficilement appréciable. Pour ailleurs, la différence statistiquement significative du taux de non réponse pour l’évaluation des connaissances sur le VIH et de l'observance des 24 dernières heures est susceptible d'induire des erreurs différentielles dans les résultats y afférents. Enfin, la collecte d'une partie des données ne se faisant pas en insu, les patients en présence de l'enquêteur pourraient par sentiment de culpabilité dissimuler leur comportement sexuel avant la découverte de leur séropositivité.

## Conclusion

Rien n'est plus certain, les personnes âgées ne sont pas à l'abri de la pandémie du VIH. Le diagnostic de l'infection par le VIH chez ces individus se fait encore tardivement, plus souvent orienté par la survenue d'infections opportunistes. Moins de 10% d'entre eux commencent le traitement avec un déficit modéré ou au stade non SIDA. Leur probabilité d’être présent dans la file active est identique à celle des plus jeunes, la moyenne de suivi dans les deux groupes étant de l'ordre de 24 mois. Ils sont autant observants que leur plus jeunes semblables. Nous pensons qu'un accent particulier devrait être mis sur le soutien psychosocial de ces patients, qui dans bien des cas ont perdu leur conjoint. Enfin, la promotion du dépistage et les programmes d’éducation sanitaire sur les risques de transmission et les moyens de prévention du VIH/SIDA devraient être renforcés au sein de cette communauté encore négligée par la stratégie nationale actuelle de lutte contre le VIH.

## Annexes

**Annexe (PDF 68Kb):** Questionnaire d’évaluation du niveau de connaissances relatives à la transmission et la prévention du VIH, aux comportements sexuels et pratiques à risque et à l'observance déclarée des 24 dernières heures
